# Cardiac involvement of *myotonic dystrophy type II* in patients with preserved ejection fraction - Detection by CMR

**DOI:** 10.1186/1532-429X-17-S1-P315

**Published:** 2015-02-03

**Authors:** Luisa M Schmacht, Julius Traber, Ulrike I Grieben, Wolfgang Utz, Matthias A Dieringer, Peter Kellman, Simone Spuler, Jeanette Schulz-Menger

**Affiliations:** 1 Cardiology, ECRC, Charité University Medicine Berlin and HELIOS Clinics, Berlin, Germany; 2 Neurology, ECRC, Charité University Medicine Berlin, Berlin, Germany; 3 Laboratory of Cardiac Energetics, National Institutes of Health, Bethesda, MD, USA

## Background

Myotonic dystrophy type II (MD2) is a genetic multisystemic disorder characterized by skeletal muscle (SM) symptoms, metabolic changes as well as arrhythmias^1^. Histopathologic changes of the SM may include fibrosis and fatty degeneration^2^. The aim of this study is to evaluate myocardial structure in preserved ejection fraction (EF).

## Methods

We prospectively enrolled 32 subjects with a genetically confirmed diagnosis of MD2. Exclusion criteria were known cardiac diseases and contraindication for CMR. We assessed left-ventricular (LV) volumes, mass and function applying state of the art cine imaging using a 1.5 T Scanner. Late enhancement imaging (LGE; slice thickness (sth) 7 mm) was performed to detect myocardial fibrosis 10 minutes after injection of gadoteridol (0.2 mmol/kgbw). We applied T1 Mapping based on MOLLI (TI native 211 ms, TI post-contrast 281 ms, TE 1.08 ms, sth 6 mm) before and 15 minutes after contrast application and assessed resultant extracellular volume fraction (ECV). Fat-water-separated imaging^3^ (GRE, TR 944.80 ms, TE 1.53-8.22 ms, sth 6 mm) was performed to identify myocardial fat deposits. Furthermore, we used ^1^H magnetic resonance spectroscopy (MRS) (TR 1600 ms, TE 35 ms, septal voxel 20 x 15 x 6 mm³) to quantify myocardial Triglycerides (MTG). Data were analyzed using cvi^42^ and standard line-fitting procedure.

## Results

**Figure 1:**
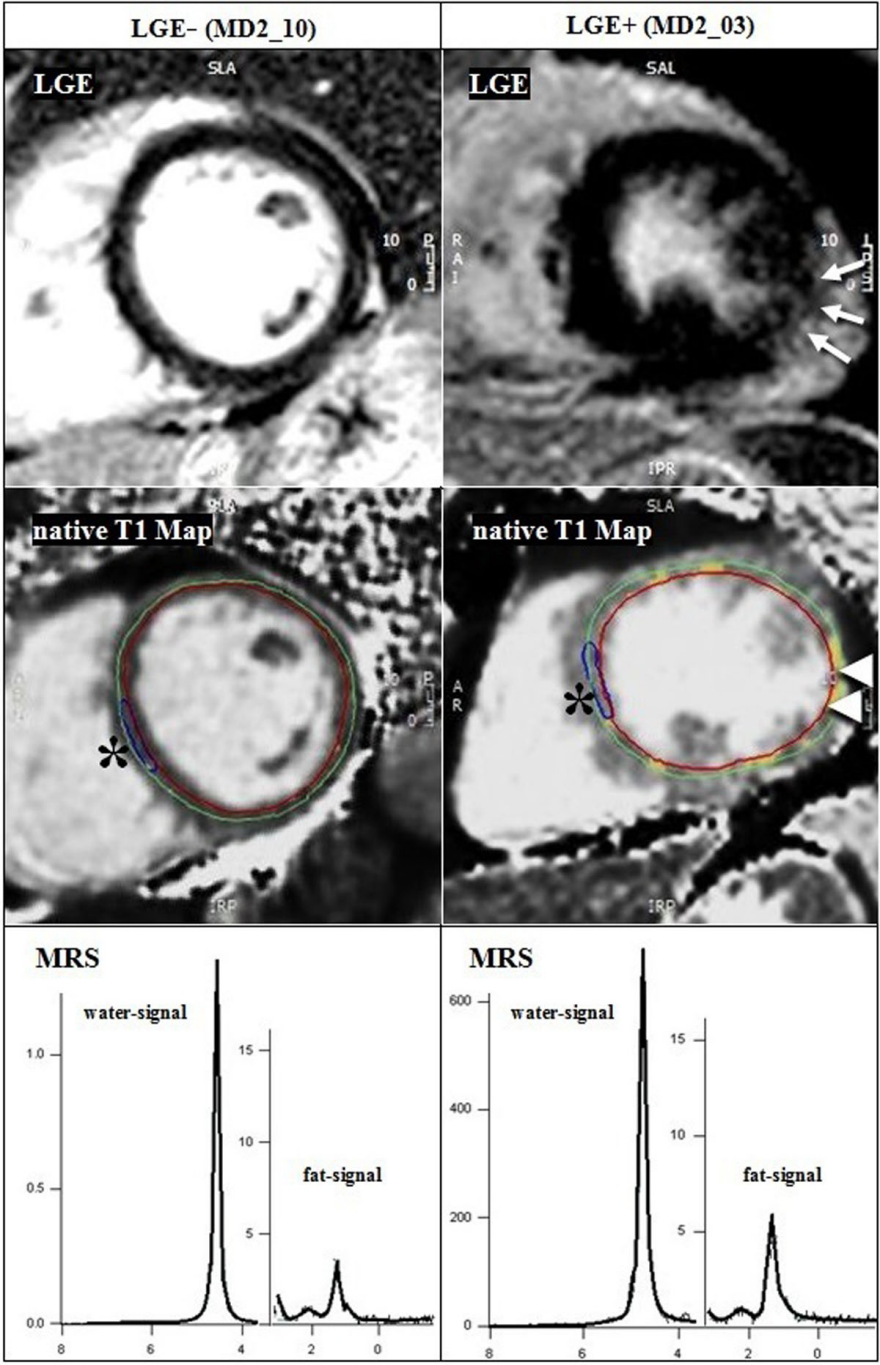
**Image findings in LGE negative (LGE-, on the left) and positive (LGE+, on the right) patients.** On top the LGE images, followed by native T1 Maps and MRS spectra. Arrows: LGE+; colored voxels (arrowheads): T1 values + 2 standard deviations to the reference myocardium (stars)

**Figure 2:**
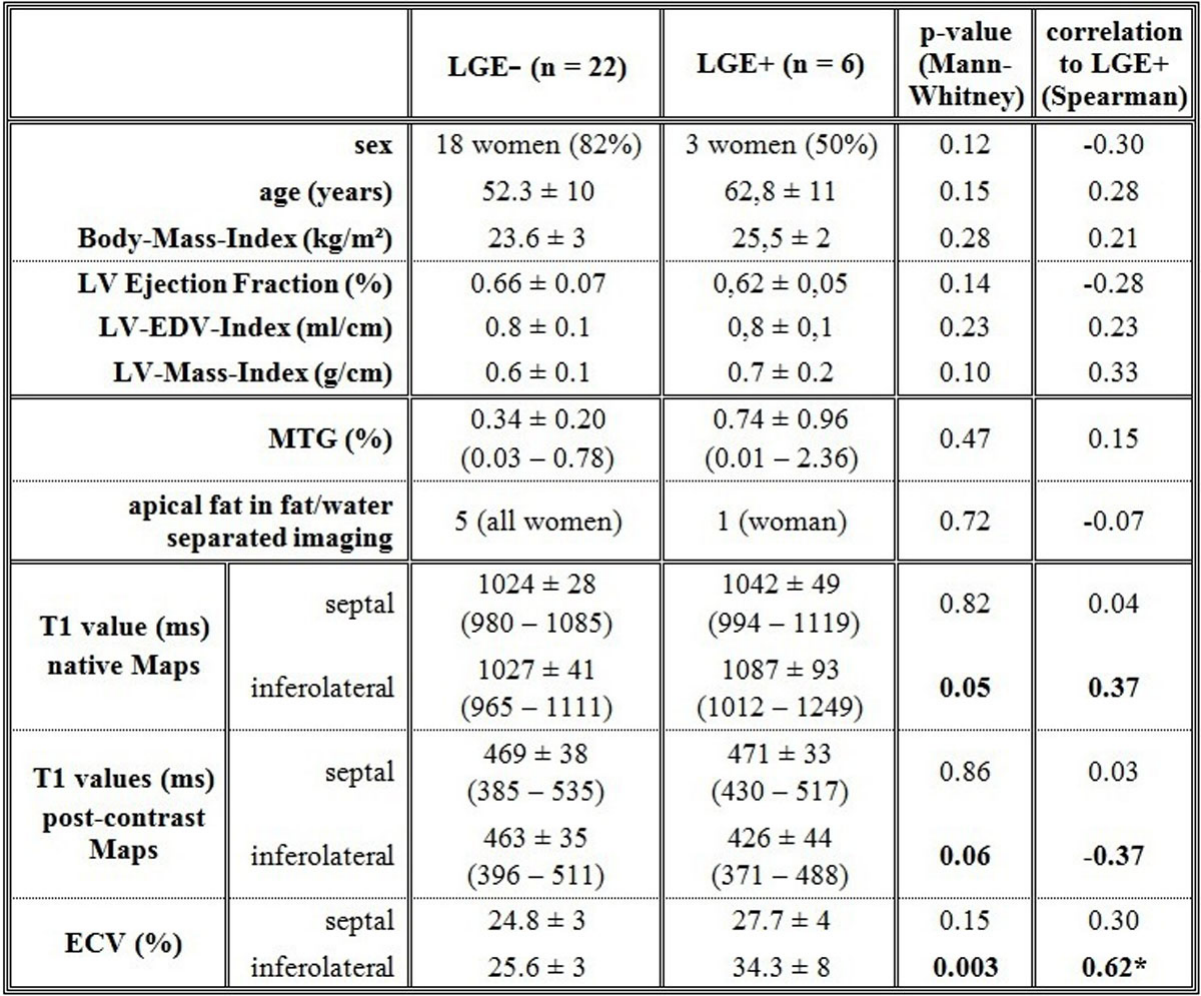
**Comparison of LGE negative (LGE-) and LGE positive (LGE+) subjects.** The inferolateral extracellular volume fraction (ECV) was significantly different in these two groups. MTG: Content of myocardial triglycerides measured by magnetic resonance spectroscopy. * The correlation to LGE+ was significant (p < 0.01)

26 data sets were totally completed (age 53.8 ± 11 y, LVEF 65 ± 0.6 %, 19 women). None of the patients had wall motion abnormalities. LGE was detectable in 6 of 28 subjects (LGE+; 3 women); the location was mostly subepicardial inferolateral basal (Figure 1). In case of LGE+, the T1-values of the enhanced regions were significantly different to remote myocardium in both the native (p = 0.03) and the post-contrast maps (p = 0.03). ECV and T1 values of the remote myocardium were not different between LGE+ and LGE negative (LGE-) groups besides to the inferolateral located ECV (25.6 % vs. 34.3 %, p = 0.03) (Figure 2). Fat deposits were noticeable in 6 of 30 subjects (all women, one with LGE+) in the apical portion of the interventricular septum. The content of MTG in LGE+ and LGE- subjects was not significant different (0.34 % vs. 0.74 %, p = 0.47).

## Conclusions

Despite preserved LVEF we could detect myocardial injury in patients with myotonic dystrophy type II. Already native T1-values were increased in case of subepicardial fibrosis compared to remote myocardium. To conclude, this is the first study constituted that CMR is feasible to detect subclinical myocardial manifestations in MD2.

## Funding

N/A.
